# Identifying Social Network Conditions that Facilitate Sedentary Behavior Change: The Benefit of Being a “Bridge” in a Group-based Intervention

**DOI:** 10.3390/ijerph17124197

**Published:** 2020-06-12

**Authors:** Sabina B. Gesell, Kayla de la Haye, Evan C. Sommer, Santiago J. Saldana, Shari L. Barkin, Edward H. Ip

**Affiliations:** 1 Department of Social Sciences and Health Policy, Wake Forest School of Medicine, Winston-Salem, NC 27101, USA; 2 Department of Implementation Science, Wake Forest School of Medicine, Winston-Salem, NC 27101, USA; 3 Department of Preventive Medicine, University of Southern California, Los Angeles, CA 90007, USA; delahaye@usc.edu; 4 Department of Pediatrics, Division of Academic General Pediatrics, Vanderbilt University Medical Center, Nashville, TN 37232, USA; evan.c.sommer@vumc.org (E.C.S.); shari.barkin@vumc.org (S.L.B.); 5 Department of Biostatistics and Data Science, Wake Forest School of Medicine, Winston-Salem, NC 27101, USA; ssaldana@wakehealth.edu (S.J.S.); eip@wakehealth.edu (E.H.I.)

**Keywords:** sedentary behavior, obesity, clinical trials, behavior strategies, Hispanics

## Abstract

Using data from one of the first trials to try to leverage social networks as a mechanism for obesity intervention, we examined which social network conditions amplified behavior change. Data were collected as part of a community-based healthy lifestyle intervention in Nashville, USA, between June 2014 and July 2017. Adults randomized to the intervention arm were assigned to a small group of 10 participants that met in person for 12 weekly sessions. Intervention small group social networks were measured three times; sedentary behavior was measured by accelerometry at baseline and 12 months. Multivariate hidden Markov models classified people into distinct social network trajectories over time, based on the structure of the emergent network and where the individual was embedded. A multilevel regression analysis assessed the relationship between network trajectory and sedentary behavior (N = 261). Being a person that connected clusters of intervention participants at any point during the intervention predicted an average reduction of 31.3 min/day of sedentary behavior at 12 months, versus being isolated [95% CI: (−61.4, −1.07), *p* = 0.04]. Certain social network conditions may make it easier to reduce adult sedentary behavior in group-based interventions. While further research will be necessary to establish causality, the implications for intervention design are discussed.

## 1. Introduction

Both theory [1,2,3,4] and empirical data [5,6,7,8,9,10,11] suggest that social networks can help diffuse both positive and negative health behaviors through groups. The architecture of social networks affects how new information, norms, and behaviors diffuse through groups. An individual’s position in a social network (e.g., being central and highly connected or peripheral and relatively isolated) influences what information or behaviors they are exposed to and adopt. The social network’s structure—whether densely or sparsely connected, or clustered into sub-groups—also influences the diffusion of information and behaviors. Overall, as new health information and behaviors are learned by someone in a social network, the network architecture will determine the flow of those ideas and behavior norms to others in the network. See Figure S1 for a conceptual model showing how connections between people influence health.

Given that social networks have been found to influence health behaviors in free-living conditions, there is value to understanding how social network characteristics are associated with behavior change in group-based interventions. Research has shown that when groups of strangers are brought together to participate in a behavior change intervention, they form social ties in predicable patterns, forming an emergent social network [12]. Network theory suggests that these social ties can measurably influence the desired behavior change, depending on complex combinations of the group network structure, someone’s position within the network, and the target behavior [3,4]. However, few studies have examined how these new networks support or undermine program outcomes.

In the US, adults spend approximately 7.7 h per day being sedentary [13]. Sedentary behavior is clearly associated with poor health outcomes [14]; identified as an independent risk factor for a variety of chronic health conditions, ranging from cardiovascular disease, cancer, type 2 diabetes, to depression and anxiety [15,16]. Evidence on effective intervention strategies to reduce adult sedentary behavior is growing, and new behavior change techniques are being sought [17]. Social network-informed interventions may be a new way to reduce sedentary behavior by leveraging social influences to support behavior change.

The goal of this paper was to examine the social network conditions that developed when groups of strangers were brought together to participate in a behavior change intervention, and to determine whether those network conditions are associated with their sedentary behavior. This study focuses on (1) understanding the extent to which particular characteristics of the social networks that formed among groups of parents while participating in an intervention were associated with follow-up sedentary behavior; and (2) identifying a leverage point for novel interventions that intentionally use social networks to reduce the amount of time parents spend sedentary. To do this we conducted a secondary analysis using data collected during a community-based healthy lifestyle intervention that was delivered in small, consistent groups to determine whether the group network structure and participants’ positions within their group influenced their sedentary behavior. Applying a multivariate hidden Markov model (MHMM) to social network data collected over the 12-week intervention, individuals were classified into unique network trajectories with different combinations of network characteristics over time. Within the context of an intervention that promoted healthy behaviors, we hypothesized that the individuals who were highly connected in densely connected groups would have a greater reduction in sedentary behavior compared to those who were isolated in sparsely connected groups.

## 2. Materials and Methods

### 2.1. Sample

Data were collected in a community-based healthy lifestyle intervention study to prevent pediatric obesity in low-income, predominantly Hispanic, families (Growing Right Onto Wellness, GROW) [18]. Families were eligible to participate in the GROW study if they met the following criteria: (1) Primary caregiver ≥ 18 years; (2) Child 3–5 years; (3) Child overweight but not obese (BMI percentile ≥ 50 and <95 based on US Centers for Disease Control and Prevention standardized growth curves) [19]; (4) English or Spanish-speaking; (5) Parental commitment to participate throughout the study; (6) Consistent phone access; (7) Recruitment from Nashville zip code regions within 8-km radius of participating community centers; (8) Participant qualification for at least one service for underserved populations (e.g., Medicaid, Special Supplemental Nutrition Program for Women, Infants, and Children) [20].

In the original study, 610 low-income, adult-child dyads were randomized. The adult primary caregiver was usually the mother (96.5%), and 91.1% of the adults were Hispanic. At baseline, 306 dyads were randomized to the control arm (GROW Smarter); and 304 dyads were randomized to the intervention arm (GROW Healthier) and assigned to one of 30 small groups of approximately 10 dyads. All study participants received the GROW Smarter school readiness curriculum. Participants in the intervention arm also received a lifestyle intervention that was delivered to each of the 30 groups in person for 12 weekly 90 min-sessions (an “intensive group phase”) and followed by nine monthly phone call coaching sessions from their interventionist, to reinforce healthy goals set by participants. Refer to our published work for all intervention details [20]. Intervention and control group activities and data collection were done separately, to minimize contamination. The present study analyzed data from adults in the intervention arm who completed social network surveys at least once during weeks 3, 6, and 12 of the intensive group phase, and who had valid sedentary behavior or BMI data at 12-month follow-up (N = 261/304), immediately following the nine monthly coaching sessions. The regression analysis used N = 187/261 cases. 

### 2.2. Intervention

The GROW Healthier lifestyle intervention was delivered in small groups, with participants consistently attending the same group. The group classes specifically promoted healthy lifestyle behaviors, including decreasing sedentary behavior. For example, behavior change techniques to reduce sedentary behavior included setting aside mobile phones for a set period of time and focusing entirely on playing with their children instead. The intervention also introduced participants to community recreation centers, by holding all sessions in the recreation center, giving participants a tour of the facilities and available classes, teaching families how to use free facility spaces such as the outdoor and indoor walking tracks, developing and providing family-based group physical activity programs outside of the intervention sessions, hiring a Spanish speaker for the front desk to welcome visitors and answer questions, printing materials in Spanish, and providing participants a free family membership so that up to five members of the family could participate in activities.

The intervention was developed to build new social networks by using social network measures and diagnostics to guide the intervention implementation [21] and to intentionally create peer-to-peer interaction to spread new behaviors within small groups of parents [2]. To do this, the social networks that emerged in the groups were monitored at multiple timepoints, and the interventionist was given feedback about how to adjust interpersonal interactions to encourage group cohesion [21]. The Social Networks Diagnostic Tool that was developed and used for the monitoring and intervening on group dynamics is described in detail elsewhere [21].

We computed multiple network diagnostics (isolates, density, centrality, subgroups, transitivity, cohesion) [22], in order to understand the social network features within each small intervention group by the midpoint of the intensive group phase (week 6 of the 12-week intensive group phase). Using pre-determined thresholds [21] defined in the Social Networks Diagnostic Tool, we then created an action report for the interventionist with concrete recommendations (e.g., connect Participant 1 with any of these four group members; make sure Participants 2 and 3 do not form a separate subgroup) tailored to the group dynamics at week 6. Interventionists were instructed to use the action report recommendations during the remaining sessions (week 7–12) to increase group cohesion. Fidelity was assessed in more than 10% of all sessions and was high, at 99% adherence to protocol.

### 2.3. Data Collection

The written informed consent process occurred in participants’ language of preference (Spanish or English) by a trained bilingual data collector. The Vanderbilt University Medical Center Institutional Review Board (IRB No.120643) and an NHLBI (National Heart, Lung, and Blood Institute)-appointed Data and Safety Monitoring Board approved the protocol and routinely evaluated participant safety and protocol adherence. Data were collected in Nashville, USA, from June 2014 to July 2017.

### 2.4. Measures

#### 2.4.1. Sedentary Behavior

Mean daily minutes of sedentary behavior were collected at baseline and 12-month follow-up using GT3X+ accelerometers (ActiGraph, Pensacola, FL, USA). Sedentary behavior (minutes) is operationalized as occupational or leisure time sitting, reclining, or viewing screens. Matthews’ validated cut points were used to define sedentary behavior (≤100 counts/min) for adults [13,23]. Adults were instructed to wear the monitor on their hip continuously for 7 complete days, including sleeping and during bathing and swimming. Participant wear time (minutes) was used to determine whether participants met validity criteria. The minimum valid wear time required 4 days (3 weekdays and 1 weekend day) of at least 6h of wear time, between 5:00 a.m. and 11:59 p.m. However, participants could, and often did, wear accelerometers for much longer. Participants failing to meet the minimum criteria were asked to repeat the ActiGraph measurements. Sedentary time was not standardized based on total wear time; rather, statistical models included mean daily wear time (minutes) as a covariate, thereby adjusting results for individual differences in wear time (see Data Analysis section).

#### 2.4.2. Intervention Group Social Networks

Social network data were collected at three timepoints (weeks 3, 6, and 12) during the 12 weeks of the intensive group phase of the intervention. Advice seeking was the social relationship assessed within each intervention group, measured with the survey question: “In your GROW group, who would you go to outside of sessions for advice on making your family healthier (like being more active, eating healthier, and getting more sleep)?” This question was selected because social networks in which one discusses “health matters” (not just “important matters”) predict health and health services-related outcomes [24]. Advice seeking from others denotes trust, and can reflect a hierarchy of expertise and knowledge, which are important components of interpersonal influence [25]. We measured advice-seeking interactions outside group sessions to capture stronger personal ties than those that might exist only in-session. Participants could list up to 7 group members, out of the typical 10 that were in each group. 

The intervention assistant read the network question aloud. Participants received a sheet with photos and names of the other group members and stickers to place on the photos of individuals from whom they sought advice. This aided recall reduced possible measurement error from low literacy, partial names, and similar or identical names. Data were collected at the beginning of the group sessions; absent participants were asked to complete the surveys at subsequent sessions (week 4 and week 7). These data were used to generate advice-seeking networks, represented as directed adjacency matrices, for each intervention group at weeks 3, 6 and 12.

#### 2.4.3. Data Analysis—Network Statistics

##### Network Statistics

A network consists of individuals (nodes) and the relationships (ties) connecting them, and it is represented as a directed matrix where a directed tie between each pair of nodes = 1 if advice-seeking is reported, and = 0 if there is no advice tie. Statistics that summarize the structural characteristics of the social networks were computed for each group at all three timepoints. The five network statistics included: individual-level network statistics: (1) indegree (the number of incoming ties to an individual), (2) outdegree (the number of outgoing ties from an individual), and (3) betweenness centrality (the number of tie paths passing through an individual); and group-level network statistics: (4) density (proportion of potential connections in the group that are observed connections) and (5) transitivity (triad structures that represent “a friend of my friend is my friend”; a facet of social balance) [26] (see File S1 for detail).

MHMM [27,28,29,30,31], which can be considered a longitudinal version of latent class analysis, was used to analyze the network statistics at each timepoint, and to classify every participant into one of several network states, with each state characterized by a unique latent profile of network statistics (see File S2 for details). The five network statistics (three individual- and two group-level network statistics described above) were used simultaneously to identify the best number of distinct latent network states to represent the data based on the following: the Bayesian information criterion (BIC), a goodness-of-fit index; how many members were in each network state (because states with few members may not be robust); interpretability; and practical usefulness. MHMM generated a pattern of network states for each participant across the three timepoints and provided an estimate of the likelihood of an individual transitioning from one state to another over time. Because of the large number of possible network state patterns over time, a heuristic was used to classify specific patterns into a smaller number of distinct “network trajectories”. The network trajectory variable essentially grouped participants who had similar social network experiences over the intervention period, and it was used as they key independent variable to predict sedentary behavior at 12-month follow-up in a multilevel regression analysis. 

Multilevel analysis was appropriate for this secondary analysis because the data were nested (i.e., adults were organized into groups that received the intervention together). In the multilevel regression, the first level represented individuals, the second represented the 30 small groups, and the dependent variable was mean daily time spent in sedentary behavior at 12-month follow-up (minutes). Besides the key independent variable of network trajectory, other covariates at the first (individual) level included baseline age (years), sex, pregnancy status (weeks pregnant and weeks since birth), mean daily accelerometer wear time at 12-month follow-up (minutes), and baseline mean time spent in sedentary behavior (minutes). The inclusion of the latter two covariates was important because it allowed for the relationship between network trajectory and follow-up sedentary time to be assessed, while adjusting for potential individual-level differences in follow-up wear time and prior sedentary behavior. Second-level (group) explanatory variables included location (one of two recreation centers) and group size. The resulting network states, trajectories, and decision rules used to define them appear in the Results. The MHMM was estimated using Matlab-based software v9.3 (The MathWorks Inc., Natick, MA, USA) [32], and the multilevel regression model was implemented using SAS v9.4 (SAS Institute Inc., Cary, NC, USA) [32].

## 3. Results

### 3.1. Baseline Characteristics

The analytic sample for the current study included 261 adult primary caregivers and reflected the total sample from the original study [18]. Among the analytic sample, 98.5% were female, 91.2% were Hispanic, and 55.6% had a total household income < $25,000. Mean (SD) age was 32.5 (6.2 years). Mean (SD) body mass index was 29.9 kg/m^2^ (6.4). At baseline, the mean (SD) adult sedentary behavior was 475 (131) min/day. At the 12-month follow-up, it was 442 (132) min/day. Mean adult wear time at baseline was 1001 (156) min/day, and 97.9% (238/243) had at least 10 h of wear time. At 12-month follow up, wear time was 962 (173) min/day, and 96.5% (191/198) had at least 10 h of wear time. 

### 3.2. Social Networks

#### 3.2.1. Network States

Thirty-four percent (34%) of participants did not seek advice from anyone, 22% sought advice from one person, and 44% sought advice from two or more people. Seven participants listed the maximum of seven possible advice nominations.

**Figure 1:**
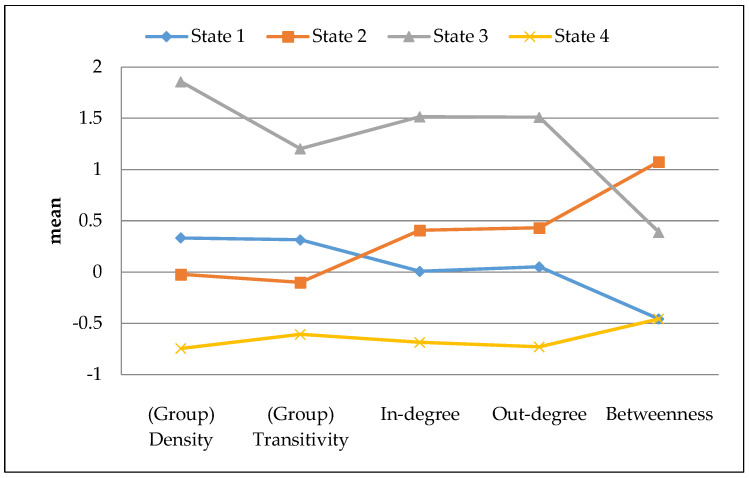
Distribution of the standardized network statistics. Note: X-axis represents social network statistic, Y-axis represents standardized mean value.

**Figure 2:**
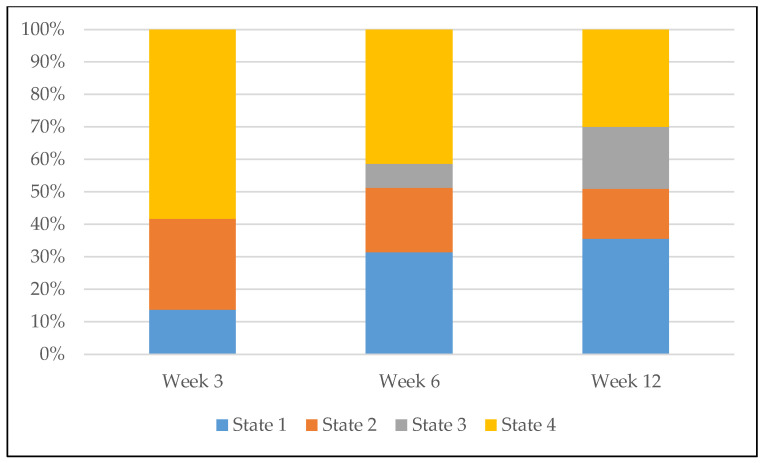
Prevalence of the four latent network states over time. Note: X-axis represents timepoint, Y-axis represents prevalence (%).

The BIC values from the MHMM analysis appear in Table S1. BIC values differed little among models with four, five, and six states. The four-state solution (Figure 1) was the most parsimonious and included well-separated and interpretable states, each with adequate sample representation (Figure 2) (see File S3 for detail).

A decision was therefore made to adopt the four-state model. Table 1 shows the transition probabilities—the likelihoods of a randomly selected individual staying in the same state or moving from one state to another state between two consecutive timepoints.

#### 3.2.2. Network Trajectories

Heuristics grounded in social network theory [2,4] were developed to classify patterns of network states that participants occupied across timepoints into four discrete trajectories (see Table S2 for detail). The betweenness network statistic is important for the diffusion of behavior, because it facilitates the ‘bridging’ of information to less-connected nodes of the network. Therefore, adults who occupied the state with the highest degree of betweenness (i.e., State 2) at any of the three timepoints were coded into a network trajectory labelled Bridge (N = 103/261, 39.5%). Within the context of a health behavior intervention, there are disadvantages to being disconnected from the group network and in a more sparsely connected network. Therefore, participants who were in the more isolated and sparsely connected State 4 at all three timepoints were assigned to the trajectory labelled Isolated (N = 67/261, 25.7%). Finally, the remaining network state patterns were classified as either Average or Popular network trajectories, depending on the characteristics of the state most frequently occupied during the three timepoints: 

Average (N = 69/261, 26.4%): Participants in States 1 or 4 for one or more of the three timepoints, but who were never in State 2 or State 3, held average positions in average networks. 

Popular (N = 22/261, 8.4%): Participants in State 3 at weeks 6 or 12 had many ties that they sent to and received from other intervention group members, but had lower betweenness centrality (and so were not bridging connectors in the group). Adults moving into this state largely came from States 1 and 2, and tended to remain in State 3 (i.e., they stayed popular once becoming so). Table 2 summarizes the characteristics of these four groups.

### 3.3. Predicting Sedentary Behavior from Network Trajectory

Using multilevel regression analysis, network trajectory was used to predict mean daily time in sedentary behavior at the 12-month timepoint, controlling for relevant covariates at individual and group levels. The Isolated network trajectory was used as the reference in the regression analyses, because these participants were likely to have the least positive benefit from their group social network. We found that being in the Bridge network trajectory during the 12-week intensive group phase of the intervention predicted an average reduction of 31.3 min in mean daily sedentary behavior 9 months later (at the 12-month follow-up), versus those in the Isolated network trajectory [95% CI: (−61.4, −1.07), *p* = 0.04, Table 3].

## 4. Discussion

### 4.1. Predicting Sedentary Behavior

We hypothesized that, within the context of an intervention, adults who were popular (had many ties/high indegree) would have reduced sedentary behavior at follow-up, theorizing that those being sought out for advice may be the most successful at adopting health behaviors being promoted in the intervention. However, we did not find evidence to support this hypothesis.

Instead, adults with the Bridge trajectory (high betweenness centrality at some point during the 12 weeks, in otherwise ‘average’ networks) had a statistically significant reduction in mean daily sedentary behavior of 31.3 min [95% CI: (−1.07, −61.4), *p* = 0.04] at the 12-month follow-up, compared to those with the Isolated trajectory (i.e., participants with few ties and in less densely connected networks), even after adjusting for baseline sedentary behavior.

Almost 40% of participants were classified as occupying the high-betweenness State 2 at one or more timepoints during the intervention, with 6% of participants in this state at week 3 and remaining in this state at the following two timepoints; and approximately 34% cycling in and out of this state during the 12 weeks.

No detectable relationships emerged among the other trajectories and sedentary behavior versus the Isolated trajectory.

To date, there is insufficient evidence to determine a public health target for total time spent in sedentary behavior, or to recommend how often sedentary time should be interrupted with physical activity each day [17,33,34]. The reduction in sedentary behavior of 31.3 min/day (3.7 h/week) among Bridges is clinically meaningful, because replacing any amount of sitting time with even light-intensity physical activity reduces the risk of all-cause mortality and cardiovascular disease in adults [34].

Bridges in social networks tend to hold advantageous social positions because they connect and broker information and advice flow through the network. This social position can provide access to more diverse, and possibly novel, types of information and knowledge, and richer social capital [35,36]. Bridges in this intervention may have been exposed to more diverse types of advice and resources from different clusters in their groups, and may have synthesized this information in a way that supported healthy behavior change, versus those socially isolated within groups. Unmeasured individual characteristics, such as agreeableness, openness to new experiences, or other personality or social traits (i.e., willingness to adhere to an intervention), may predict classification as a Bridge [37] and/or success in reducing sedentary behavior. Nonetheless, while Bridges had fewer advice ties sent or received (reflecting a less prominent social status), compared to adults in the Popular network trajectory, the bridging social network role appears to benefit the individuals themselves, in terms of their ability to change their own health behavior, relative to Isolates.

To the best of our knowledge, this is the first study that examines how a complex array of social network characteristics were associated with objectively measured sedentary behavior within a behavioral intervention that deliberately sought to foster new ties. Additionally, this finding is reinforced by previous research [38], which found that social isolation—not having any network connections—was associated with higher odds of physical inactivity, measured as sedentary and low intensity activity with the International Physical Activity Questionnaire (IPAQ) [39].

### 4.2. Limitations

Given our modest sample size, we opted to select a small number (in this case, five) of salient but distinct network features expected to relate to health behavior change [4], based on the prior literature. A different constellation of network statistics might yield different results. The heuristics we used to categorize trajectories was informed by social network theory [1,2,3,4] and allowed us to classify participants into well-represented groups. This sufficed for our objective, but may not be the optimal clustering approach. The data had some degree of missingness, but results were based on the 86% (N = 261/304) of intervention participants with enough data for analysis. Analyzing objectively measured sedentary behavior among low-income minority primary caregivers is an important step in addressing health equity and increasing representation with the body of scientific research. However, these sedentary behavior results may not generalize to other health behaviors or different populations. While intervention and control group activities took place separately, participants lived in the same region and connections could have existed or formed over time between individuals in the two groups, despite efforts to avoid contamination. Support for causal inference is limited by three issues: (1) it was not possible to randomize assignment to network trajectory because it is a new construct and it is based on participant experience after the intervention has already begun; (2) it was not possible to make a comparison involving the control group because they did not meet in small groups or provide advice network data; and (3) this was a secondary analysis that was not pre-specified. Several commonly used statistical models for social networks, such as the stochastic actor-based model [40], were attempted, but are poorly suited for modeling newly emerging networks on multiple small groups where network ties change substantially over time, as is the case in our data. Those models are not well suited to test how exposure to different combinations of individual and group network structures together predict individual outcomes, while the MHMM allowed us to model more holistic and changeable network states that participants experienced over time.

### 4.3. Implications for Intervention Design

Many behavioral interventions are delivered in group settings, but few studies have explicitly assessed how group interactions evolve to support or undermine program outcomes. Our study addresses this gap in the literature and suggests that participants who were exposed to a specific network typology—occupying a bridging position in a relatively connected group network—may reduce sedentary behavior within a behavior change intervention organized by groups. Capturing social network data and using MHMM and a two-level analysis during the implementation of the group intervention permits several options for supporting program outcomes. While further research will be necessary to establish causality, these results have implications for intervention design. Interventions aimed at reducing sedentary behavior might (1) identify “bridges” early and later task them with providing advice to support the desired behavior change across the group (i.e., serve as champions); (2) regularly monitor the group network structure to identify disconnected subgroups, and promote new interactions that would result in participants adopting a bridging role between these disconnected groups; and/or (3) identify opportunities for more people to cycle in and out of being bridges during the intervention. These strategies may amplify intervention effects in a group setting. While further research will be necessary to establish causality, fostering the creation of Bridge trajectories within new social networks may be a promising approach to reducing adult sedentary time in behavioral interventions. 

### 4.4. Future Research Directions

Future research could take several approaches. To establish causality, one approach would be to add a social network-building component (focused on building new advice ties in particular) to an intervention that has been shown to reduce sedentary time, and compare outcomes for groups randomized to receive the standard program vs. groups exposed to the program with the new network component. The only difference between groups would be whether the intervention sought to alter the participant’s social network. Based on the findings from this study, we would recommend that the interventionist use a social network diagnostics tool (such as the one described here [21]) several times during the program, to measure and visualize the structure of the intervention group advice network. Based on the observed network structures, the interventionist could guide the study participants to form new advice ties with specific individuals that would position them as a “bridge” in the group, and attempt to cycle multiple participants in and out of this beneficial position during the course of the intervention. 

Another approach would be to build new datasets of intervention group social networks and accelerometry data, with a greater number of data collection timepoints, that are more temporally dense. Stochastic actor-based models [40], implemented in the R (R Foundation for Statistical Computing, Vienna, Austria) package “RSiena”, could be used to model both behavior and network change processes and disentangle selection and influence effects, allowing for a stronger inference of causality. Unfortunately, these methods were not suited to the data collected in the current study (given the sparseness of the networks and degree of network change across a smaller number of network observations), and these datasets are expensive to develop, primarily due to the cost of collecting and processing accelerometry data. 

A complimentary line of future research would be to use agent-based models, like those implemented in NetLogo [41], to run social simulations that predict future behavior from explicitly programmed, microlevel rules, to estimate decreases in participants’ sedentary behavior. Such models would be based on an assumption that network position influences changes in sedentary behavior, but it could be used to test various intervention strategies and scenarios and give estimates of their relative effect sizes. This simulation approach would be beneficial for exploring potential intervention strategies and scenarios, saving the cost and time of implementing the various iterations of these trials in the real-world.

## 5. Conclusions

Being embedded in certain social network conditions during a group-based behavior change intervention may make it easier for low-income adults to reduce their daily sitting time. In particular, seeking advice from other study participants, such that one becomes a bridge at some point during the face to face intervention (someone who connects clusters of individuals), vs. remaining isolated throughout the intervention, may enable study participants to reduce their sedentary time by 31.3 min/day (3.7 h/week). In contrast, being popular (having many advice seeking connections to other study participants, but never being a bridge) or being average (average number of advice seeking connections in average networks, but never being a bridge) over time vs. being isolated did not facilitate behavior change. Social network analysis could complement traditional behavior change programs to help researchers design more effective interventions to reduce sedentary behavior.

## Figures and Tables

**Table 1:** Transition probabilities between states.^1^

Network State	State1	State2	State3	State4
State1	0.51	0.16	0.23	0.09
State2	0.32	0.40	0.21	0.08
State3 ^2^	0.21	0.00	0.79	0.00
State4	0.25	0.11	0.04	0.60

^1^ Each entry represents the probability of transition from a row state to a column state at any given timepoint (e.g., the transition probability from State 1 to State 2 is 16%). Each row sums to 100% (within rounding error). ^2^ The transition probabilities from State 3 to the other states are from week 6 to week 12 only.

**Table 2:** Characteristics of network trajectories.

Characteristic	Mean or Percentage (Isolated) N = 67	Mean or Percentage (Bridge) N = 103	Mean or Percentage (Average) N = 69	Mean or Percentage (Popular) N = 22	Mean or Percentage (Total) N = 261
Gender					
Male	2 (3.0%)	1 (1.0%)	1 (1.5%)		4 (1.5%)
Female	65 (97.0%)	102 (99.0%)	68 (98.6%)	22 (100%)	257 (98.5%)
Age (years)	32.8 (6.2)	31.7 (5.9)	32.9 (6.5)	33.9 (6.4)	32.5 (6.2)
Body mass index (kg/m^2^)	29.4 (5.9)	29.0 (6.2)	31.4 (7.0)	30.7 (6.7)	29.9 (6.4)
Race/Ethnicity					
Hispanic	62 (92.5%)	98 (95.2%)	59 (85.5%)	19 (86.4%)	238 (91.2%)
Non-Hispanic	5 (7.5%)	5 (4.9%)	10 (14.5%)	3 (13.6%)	23 (8.8%)
Household Income					
$14,999 or less	17 (25.4%)	31 (30.1%)	17 (24.6%)	6 (27.3%)	71 (27.2%)
$15,000–$24,999	21 (31.3%)	28 (27.2%)	20 (29.0%)	5 (22.7%)	74 (28.4%)
$25,000–$34,999	9 (13.4%)	15 (14.6%)	10 (14.5%)	2 (9.1%)	36 (13.8%)
$35,000–$49,999		1 (1.0%)	3 (4.4%)	1 (4.6%)	5 (1.9%)
$50,000–$74,999		1 (1.0%)		1 (4.6%)	2 (0.8%)
Don’t know	20 (29.9%)	27 (26.2%)	19 (27.5%)	7 (31.8%)	73 (28.0%)
Education					
High school incomplete	44 (65.7%)	64 (62.1%)	33 (47.8%)	17 (77.3%)	158 (60.5%)
High school degree or equivalent	23 (34.3%)	39 (37.9%)	36 (52.2%)	5 (22.7%)	103 (39.5%)
Accelerometry					
Mean daily total wear time (min)	999 (157)	1000 (166)	999 (147)	1018 (143)	1001 (156)
Mean daily moderate/vigorous physical activity (min)	45.1 (40.2)	45.4 (29.9)	44.0 (33.4)	72.6 (65.0)	47.4 (38.5)
Mean daily sedentary behavior (min)	469 (138)	470 (127)	491 (132)	461 (124)	475 (131)

**Table 3:** Multilevel regression predicting minutes of sedentary behavior at 12-month follow-up from network trajectory over the first 12 weeks of the intensive group phase of the intervention.

Effect	Level	Estimate (min/day)	Lower 95% CL	Upper 95% CL	*p*-Value
Intercept		−152.76	−315.72	10.2	0.07
Trajectory ^1^	Popular	−7.94	−51.95	36.06	0.72
	Bridge	**−31.26**	**−61.45**	**−1.07**	**0.04**
	Average	6.89	−26.57	40.34	0.68
	Isolated				
Mean daily sedentary behavior (min) at baseline		**0.33**	**0.23**	**0.43**	**<0.01**
Gender	Female	44.89	−48.38	138.17	0.34
	Male				
Age		−3.46	−5.58	−1.34	<0.01
Weeks Pregnant		0.39	−2.46	3.24	0.79
Weeks Since Giving Birth		0.17	−2.82	3.16	0.91
Average total wear time in minutes/day at 12-month follow-up		**0.51**	**0.43**	**0.58**	**<0.01**
Study Site	Recreation Site 1	−2.57	−41.82	36.68	0.9
	Recreation Site 2				
Group Size		3.03	−3.17	9.22	0.34

^1^ Reference group is the Isolated trajectory, theoretically the least beneficial state trajectory, because members are not connected to the social network, and are embedded in sparsely connected networks, and thus do not have access to the information and resources flowing through the network. Bold indicates *p* < 0.05

## Supplementary Materials

The following are available online at https://www.mdpi.com/1660-4601/17/12/4197/s1, Figure S1: Conceptual Model: How social networks affect health, File S1: Network Statistics, File S2: Multivariate Hidden Markov Models, Table S1: Goodness-of-fit (BIC) values from the multivariate hidden Markov Model analysis, File S3: Network States, Table S2: Classification of state patterns into network trajectories.
