# Supplementary material for: Identifying Social Network Conditions that Facilitate Sedentary Behavior Change: The Benefit of Being a “Bridge” in a Group-based Intervention
Source: Int J Environ Res Public Health. 2020 Jun 12;17(12):4197. doi: 10.3390/ijerph17124197 (PMC7344869; doi:10.3390/ijerph17124197)
Supplement: Supplementary file 1 [file ijerph-17-04197-s001.pdf]

Figure S1:

Conceptual Model: How social networks affect health (adapted from Berkman & Glass) [1]

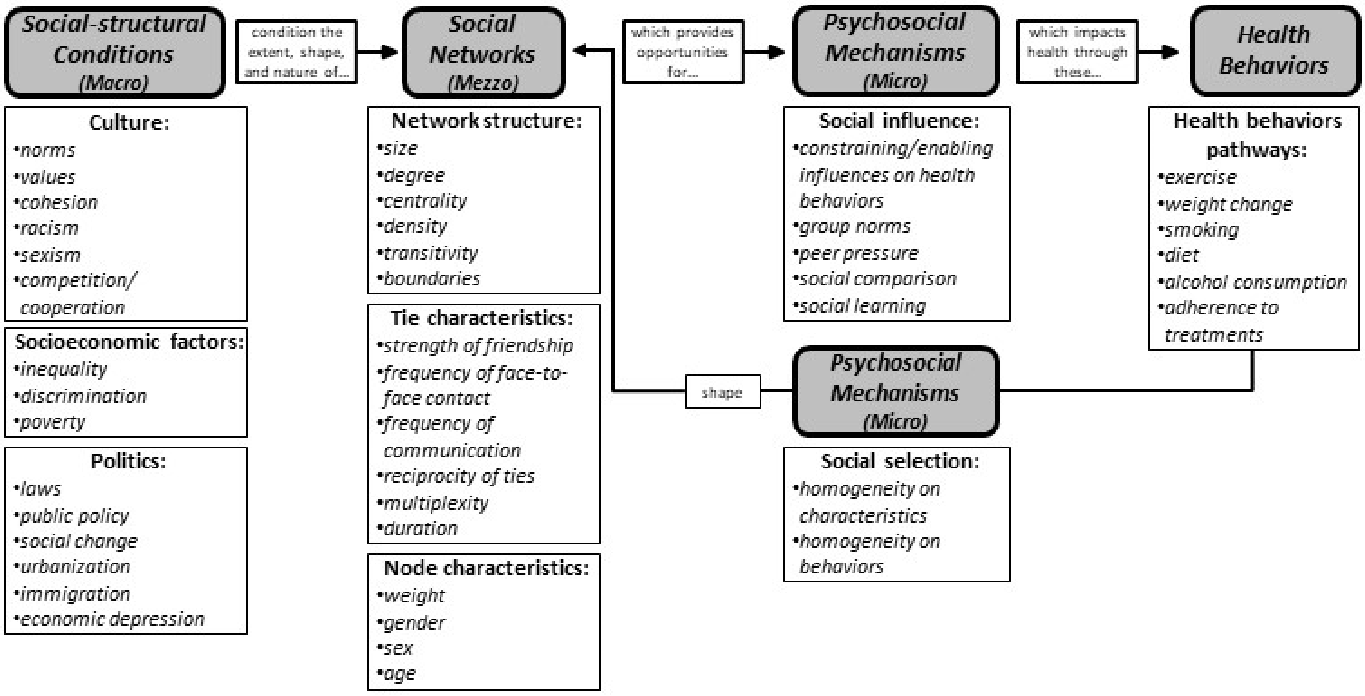

File S1:

Network Statistics

At each assessment, each intervention group's social network was defined as a matrix of relationships between each pair of group members; where 1 denotes a tie from person *i* to person *j,* and 0 denotes no tie. Network statistics at the individual- and network (group)-level were computed. MHMM [29] was then used to classify individuals with similar values on a pre-determined set of these statistics into the same latent network state (see Data Analysis section).

The following ***individual-level network statistics*** [25] were computed to capture the extent to which participants were central and connected in their group social network:

- ***Indegree centrality:*** the number of incoming ties (i.e., nominations) a participant received from other group members, normalized by the maximum theoretical indegree (group *n*-1). This measures a participant's popularity regarding providing advice to other group members.
- ***Outdegree centrality:*** the number of outgoing ties (i.e., nominations) made by a participant to other group members, normalized by the maximum theoretical outdegree (group *n*-1). This measures how actively a participant seeks advice from other group members.
- ***Betweenness centrality:*** the number of advice tie paths that travel through a participant, (akin to the idea of "six degrees of separation") normalized by the theoretical maximum given the observed size of the group. Participants with higher betweenness centrality ("fewer degrees of separation from many people") can be important conduits for advice, because they can influence the flow of information and resources through the network. They may have access to more diverse knowledge or resources from different segments of the network.

The following ***group-level network statistics*** [25] were computed to reflect the global structure of participants' social networks:

- ***Density*:** the proportion of ties observed among all group members, relative to possible ties among group members (i.e., group n*(n-1)). High density means that groups had many participants seeking advice from one another; low density reflects sparsely connected groups with little advice seeking and little potential for social influence and support.
- ***Transitivity:*** the number of triad structures (where node A is connected to node B, node B is connected to node C, and node C is connected to node A) in the network, divided by the total number of triad structures possible given the number of 3-node sets in the group. It reflects a social process where "I seek advice from you, *and* from the person from whom you seek advice", and globally represents clustering of ties into smaller sub-groups or "communities" likely to establish stronger norms and identities that could support healthy behavior change.

File S2:

Multivariate Hidden Markov Models

MHMM can be considered an extension of the latent class analysis to longitudinal setting. Like latent class and latent profile analysis, MHMM conceptualizes a causal process in which an underlying, unobserved (hidden or latent) construct is driving multiple manifest variables called indicators (hence deemed multivariate). In the case of our study, the unobserved construct is network position, whereas the indicators form the behavioral profile of an individual. The goal of MHMM is to (1) delineate clusters of individuals that have similar behavioral profiles, and (2) estimate the dynamic of change over time in which individuals may stay as a member of a specific cluster or transition from being a member of one cluster to being a member of another. The clusters are called states in the current paper. The optimal number of states for the model is derived from goodness-of-fit indexes calculated from fitting models of different number of states to the data. Transition follows the Markov assumption or the memoryless principle - namely the transition probabilities for the possible next states are only dependent on the current state and not farther back. The MHMM analysis results in a set of parameters that include the behavioral profile of each state, the initial condition of the states, and the transition probabilities between the states. It also produces a trajectory of the most probable states for each individual.

Table S1:

Goodness-of-fit (BIC) values from the multivariate hidden Markov Model analysis

# states	BIC value
2	-2809
3	-3178
4	-3614
5	-3615
6	-3680 (best)
7	-3651

File S3:

Network States

State 1. Adults in State 1 had an average number of connections (indegree centrality and outdegree centrality), although they had lower than average betweenness centrality, and they were embedded in networks of average density and transitivity. This indicates that they held average positions in average networks.

State 2. Adults in State 2 had higher than average indegree centrality and outdegree centrality and especially high betweenness centrality, indicating that they were often "bridges" among group members and had a high potential to influence others. They were embedded in networks of average density and transitivity. This indicates that they were "bridging" actors in average networks.

State 3. Adults in State 3 had lower betweenness centrality than State 2 but very high indegree centrality and outdegree centrality, and were embedded in networks with high density and transitivity. This suggests they had many ties and were in highly connected networks but were only average in "bridging".

State 4. Adults in State 4 had lower than average indegree centrality, outdegree centrality, and betweenness centrality, and they were embedded in groups with low density and transitivity. This suggests they were isolated in sparse networks.

Prevalence of the Four Latent Network States at Each Timepoint

States 1 and 4 were the most prevalent across the timepoints (**Figure 2**). The number of adults in State 4 declined from 60% at week 3 to 30% at week 12. In contrast, the number of adults in State 1 increased from 15% at week 3 to 35% at week 12. State 3 was not present at week 3, early in network formation, but 7% of adults were in State 3 at week 6, and 20% were in State 3 at week 12.

Transition Probabilities Between States

Adults in States 3 and 4 had ≥ 60% probability of staying in the same state over time, whereas adults occupying State 2 had a 40% probability of staying in that state over time (**Table 1**). Few adults transitioned into State 4.

Table S2:

Classification of state patterns into network trajectories

Rank	Percentage	Week 3	Week 6	Week 12	Trajectory name
1	25.67%	State4	State4	State4	Isolated
2	9.96%	State4	State1	State1	Average
3	8.43%	State4	State4	State1	Average
4	6.51%	State1	State1	State1	Average
24	0.77%	State4	State1	State4	Average
26	0.38%	State1	State4	State1	Average
27	0.38%	State1	State4	State4	Average
9	3.07%	State4	State1	State3	Popular
11	1.92%	State1	State1	State3	Popular
15	1.92%	State4	State4	State3	Popular
22	1.15%	State1	State3	State3	Popular
25	0.38%	State1	State3	State1	Popular
5	6.51%	State2	State2	State2	Bridge
6	5.36%	State2	State3	State3	Bridge
7	4.21%	State2	State2	State1	Bridge
8	3.83%	State2	State1	State1	Bridge
10	2.68%	State4	State4	State2	Bridge
12	1.92%	State2	State1	State3	Bridge
13	1.92%	State2	State4	State4	Bridge
14	1.92%	State4	State2	State2	Bridge
16	1.53%	State2	State1	State2	Bridge
17	1.53%	State2	State2	State3	Bridge
18	1.53%	State4	State2	State1	Bridge
19	1.53%	State4	State2	State3	Bridge
20	1.15%	State1	State1	State2	Bridge
21	1.15%	State1	State2	State2	Bridge
23	0.77%	State1	State2	State3	Bridge
28	0.38%	State2	State1	State4	Bridge
29	0.38%	State2	State2	State4	Bridge
30	0.38%	State2	State3	State1	Bridge
31	0.38%	State4	State1	State2	Bridge
32	0.38%	State4	State2	State4	Bridge
